# A four-module neural architecture for the automatic extraction and classification of causal relations in text

**DOI:** 10.3389/frai.2026.1848216

**Published:** 2026-07-23

**Authors:** Roman Taberkhan, Nurbolat Tasbolatuly, Madina Sambetbayeva, Saule Tazhibayeva, Nurmira Zhumay, Bayangali Abdygalym, Mira Kaldarova

**Affiliations:** 1 L.N. Gumilyov Eurasian National University, Astana, Kazakhstan; 2 International Science Complex Astana, Astana, Kazakhstan; 3 School of Information Technology and Engineering, Astana International University, Astana, Kazakhstan

**Keywords:** causal relation extraction, information extraction, Kazakh language, KazBERT, low-resource language, NLP, transformer models

## Abstract

This article presents a four-module system for the automatic extraction and classification of causal relationships from texts in the Kazakh language, based on the fine-tuning of the KazBERT transformer language model. The proposed architecture includes four specialized modules: recognition of lexical causality markers (Token Classification, B/I-MARKER); segmentation of cause-effect clauses (Token Classification, B/I-CAUSE · B/I-EFFECT); classification of Tv forms of markers (Sequence Classification, 16 classes); determination of the type of the marker’s syntactic construction—Model Group (Sequence Classification: SYNTHETIC/ANALYTIC/ANALYTICO-SYNTHETIC). The training was conducted using an original annotated corpus consisting of 3,223 sentences in the Kazakh language. The architecture is supplemented by a deterministic positional inversion algorithm for explanatory markers (sebebi, öitkenı, sondyqtan, etc.), which automatically restores the correct CAUSE-EFFECT argument order. Experiments have demonstrated that KazBERT outperforms the baseline models XLM-RoBERTa and mBERT: macro-F1 scores were 0.901 (tags), 0.865 (clauses), 0.884 (Tv-form), and 0.927 (construction type). The scientific novelty lies in the first publicly released four-level annotated corpus of Kazakh causal constructions, the operationalization of the established Turkological synthetic/analytic distinction—extended with a corpus-attested ANALYTICO-SYNTHETIC class—as a four-module annotation target, and a deterministic positional-inversion post-processor that corrects systematic argument-order errors for analytic markers.

## 1 Introduction

Automatic causal relation extraction (CRE) is one of the central tasks in natural language processing (NLP). The accurate identification of causal relationships enables the functioning of systems for question answering, automatic referencing, event analysis, decision support, and knowledge extraction from text corpora (Dasgupta et al., 2018; Girju et al., 2007). Despite significant progress in the field of resource languages, the Kazakh language remains largely understudied in this area. The contributions of this work are bounded as follows. We do not claim novelty for the linguistic distinction between synthetic and analytic causal subordination, which is well-established in Turkological description (see, e.g., (Johanson, 2021; Erdal, 2004; Soltanbekova and Utebaeva, 2022; Balakayev, 1959). Our contributions are:

1. A four-level annotated corpus for Kazakh causal extraction. To our knowledge, the first publicly released Kazakh corpus with simultaneous annotation at the marker, clause, Tv-form, and construction-type levels.
2. A three-way operationalization of Kazakh causal constructions for automatic annotation. While the synthetic/analytic split itself is established, its three-way operationalization—including a corpus-attested ANALYTICO-SYNTHETIC class—and its implementation as a sentence-classification target (Module M4) have not previously been formalized for Kazakh or, to our knowledge, for any Turkic language.
3. A modular four-module architecture based on KazBERT. Independent fine-tuning per subtask isolates failure modes and avoids negative transfer reported for related joint settings, while permitting per-task hyperparameter selection and module replacement.
4. A deterministic positional-inversion post-processor. The interclausal placement of analytic markers (sebebı, öitkenı, sondyqtan) is a known property of Kazakh syntax; using it as a rule-based correction layer downstream of a neural clause segmenter—with quantified error reduction—is the engineering contribution.
5. A systematic baseline comparison. First head-to-head evaluation of KazBERT, XLM-RoBERTa, and mBERT on causal extraction in any Turkic language, across all four subtasks.

Kazakh is an agglutinative Turkic language with SOV word order and a complex morphology, spoken by between 15 and 18 million people. Causal relationships in Kazakh texts are expressed through two fundamentally different mechanisms. The first is synthetic: through morphologically formed transverbal forms (Tv-forms) of the verb—participle and gerund constructions with causal suffixes (−ğandyqtan, −atyndyqtan, −ğany üşın, −ğan soñ, etc.). The second is analytical: through specific conjunctions and discursive connectors (sebebi “because,” öitkenı “since,” sondyqtan “therefore,” sol sebepti “for that reason”). There is also a mixed, analytical-synthetic type that combines both mechanisms. This three-part taxonomy is introduced in this paper for the first time in the context of automatic annotation.

Analytic markers (such as sebebı, öitkenı, etc.) that occupy an interclausal position present a particular challenge: unlike synthetic forms, they are placed after the consequent clause and before the causal clause, which reverses the standard CAUSE-EFFECT argument order. This leads to a systematic error in the direction of the causal relationship in neural network systems that are unaware of this rule. This paper proposes a deterministic positional inversion algorithm that eliminates this class of errors (Nevskaya, 2021; Devlin et al., 2019).

The introduction of KazBERT (Yeshpanov et al., 2022), a transformer model pre-trained on a Kazakh-language corpus exceeding 60 GB in size laid the groundwork for effective NLP of Kazakh texts. KazBERT consistently outperforms general-purpose multilingual models (mBERT, XLM-RoBERTa) on classification, named entity recognition (NER), and text comprehension tasks, thanks to its deep understanding of the morphological and syntactic patterns of the Kazakh language.

This article makes the following original contribution:

An annotated corpus consisting of 3,223 sentences in Kazakh has been created, with annotations at four levels: markers (MARKER), clauses (CAUSE/EFFECT), Tv-form (16 classes), and syntactic construction type (Model Group, 3 classes).

A four-module architecture based on KazBERT has been developed, covering all levels of causal analysis—from marker recognition to construction typology.

For the first time, a taxonomy of causal markers in the Kazakh language has been formalized based on syntactic construction type (SYNTHETIC/ANALYTIC/ANALYTICO-SYNTHETIC), and a corresponding classification module (M4) has been implemented.

We propose and implement a positional inversion algorithm for explanatory markers that restores the correct order of arguments in causal relationships without requiring additional training.

A systematic comparison was conducted with the baseline models XLM-RoBERTa and mBERT across all four subtasks.

## 2 Related works

### 2.1 Identifying cause and effect relationships

The task of automatically identifying cause-and-effect relationships continues to attract the attention of researchers. Traditional approaches based on lexical-syntactic patterns and rules (Girju, 2003) are highly interpretable, but do not generalize well to new domains. Statistical methods, such as SVM and CRF, have expanded their scope but remain limited in terms of semantic generalization. The shift to neural network architectures has led to significant progress.

In (Gao et al., 2019), the authors propose a BiLSTM-based approach with an attention mechanism for modeling event causality at the document level; they demonstrate a 5.3 percentage point increase in macro-F1 score by accounting for discourse structure. In (Tan et al., 2022), the problem of identifying causal relationships between events (ECI) is solved using graph neural networks; a 4.2 percentage point improvement over previous methods was achieved. The authors (Heindorf et al., 2020) created the CauseNet platform, i.e., a causality graph comprising 11 billion web pages and demonstrating the scalability of their approach. In (Yang and Mihalcea, 2022), the problem of implicit causality is formalized, requiring reasoning based on BERT. The ERGO model (Chen et al., 2022), based on a transformer with relational graphs, achieves state-of-the-art performance. The authors (Zhu et al., 2024) demonstrate that, despite the potential of prompting in LLMs, fine-tuned specialized systems outperform them on structural annotation tasks.

In order to systematize contemporary approaches to the automatic identification of causal relationships, Table 1 presents a comparative analysis of key studies. The table compares the thematic focus of the studies, the methods used, the type of model, the level of analysis, the datasets used, and performance metrics. Furthermore, the advantages and limitations of each method are highlighted, particularly in terms of multilingualism, interpretation, and suitability for low-resource languages. This comparison allows us to clearly define the proposed method’s place within the scientific landscape.

**Table 1:** A comparative analysis of the models, datasets and methodological limitations used in identifying causal relationships.

Study	Year	Task focus	Methodology	Model type	Level	Dataset/language	Performance	Strength	Gap vs. this study
Dasgupta et al.	2018	Causal relation extraction	Linguistically informed DNN	BiLSTM + features	Sentence/text	English (SemEval)	~F1:0.80	Combines linguistic + neural features	No multilingual or low-resource support
Li and Mao	2019	Causal relation extraction	Knowledge-oriented CNN	CNN (dual-channel)	Sentence/text	English/General corpora	~F1: 0.82	Uses external knowledge	Limited interpretability; no modular pipeline
Mirza et al. (CATENA)	2016	Temporal + causal extraction	Rule-based + ML	Hybrid	Event/text	English/Italian	~F1: 0.75	Joint temporal-causal modeling	Weak neural representation
Cui et al.	2022	Event causality extraction	Argument correlation modeling	Transformer-based	Event-level	English	~F1: 0.85	Captures event dependencies	Event-centric, not clause-level
Wang et al.	2024	Document-level ECRE	Knowledge-guided QA	Transformer + QA	Document/event	English	SOTA	Strong reasoning at document level	High complexity; not interpretable
Özateş et al.	2022	Causal relation annotation	Transformer-based models	BERT variants	Sentence/text	Turkish	~F1: 0.78	First Turkish causal dataset	Limited morphological diversity vs. Kazakh
German NLP studies	2020–2023	Causal relation detection	Transformer + rule hybrid	BERT/RoBERTa	Sentence/text	German	~F1: 0.80–0.85	Strong syntax modeling	Mostly rule-dependent; limited generalization
French NLP studies	2021–2023	Discourse causality	Transformer-based	CamemBERT	Sentence/discourse	French	~F1: 0.82	Strong discourse modeling	Limited clause-level annotation
Russian NLP studies	2020–2024	Causal extraction	RuBERT/DeepPavlov	Transformer	Sentence/text	Russian	~F1: 0.83	Good morphological modeling	No unified taxonomy of causal types
This Study	2026	Kazakh causal extraction and classification	Four-module KazBERT + inversion rule	Transformer (KazBERT)	Clause/text	Kazakh (3,223 sentences)	F1: up to 0.927	Modular, interpretable, linguistically grounded, low-resource	Future: discourse-level modeling, joint learning

**Figure 1:**
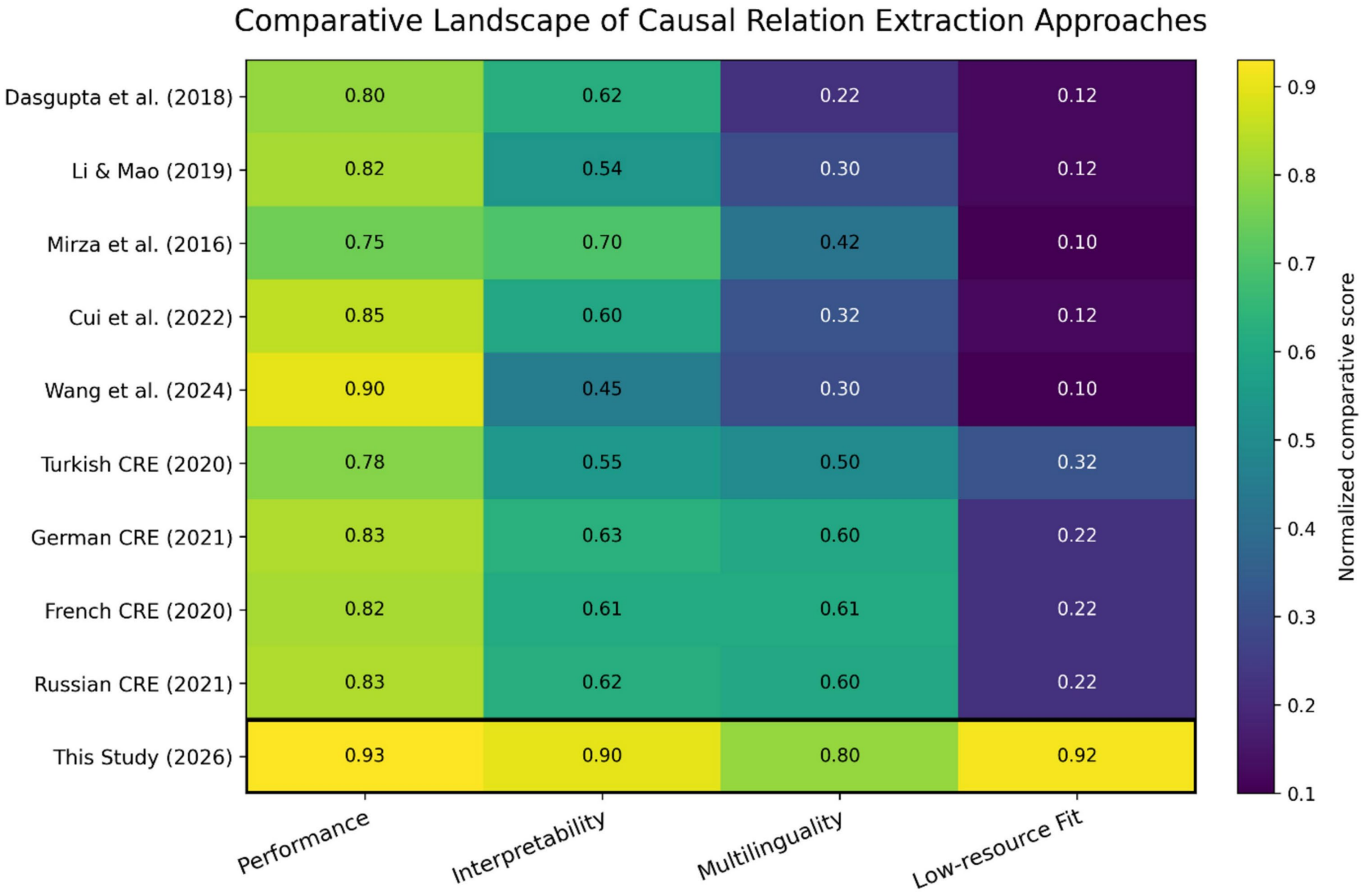
A comparative analysis of methods for identifying causal relationships, based on key criteria: productivity, interpretability, multilingualism and suitability for use in resource-constrained environments.

As shown in Table 1 and Figure 1, most existing approaches achieve high performance only on individual metrics. Whilst some models demonstrate high accuracy, they are difficult to interpret, whereas other methods better account for event structure but are primarily geared toward languages with extensive resources. The proposed method differs from these and combines, within a single system, properties such as high performance, modular interpretability, and a suitability for morphologically complex, low-resource languages. This suggests that the four-module architecture and the positional inversion mechanism based on KazBERT are an effective solution for identifying and classifying causal relationships in the Kazakh language.

### 2.2 NLP for Kazakh and Turkic languages

The Kazakh language is characterized by long chains of affixes, rich verb inflection, and non-standard word order (Makhambetov et al., 2013). The publication of KazBERT (Yeshpanov et al., 2022) marked a turning point: the model significantly outperformed XLM-RoBERTa and mBERT on tasks involving named entity recognition (NER), sentiment classification, and text comprehension in the Kazakh language. At the same time, resource bases are being expanded: NER corpora (Khassanov et al., 2021), news datasets, multi-task benchmarks (Conneau et al., 2020). Despite these achievements, the task of causal analysis for the Kazakh language remained unresolved prior to this study, both in terms of resource availability and modeling.

With regard to Turkic languages as a whole, there are only a few studies on CRE. While the general applicability of transformer models has been demonstrated for Turkish (Özateş et al., 2022), the specific features of causative constructions in Kazakh (16 distinct types) are significantly more complex than those in Turkish, and the presence of three types of causative syntactic constructions has no direct parallels in the existing literature.

### 2.3 Multitasking training and modular pipelines

Multi-task learning (MTL) allows for the joint optimization of several related tasks (Crawshaw, 2020). When dealing with tasks that have different types of outputs (tokenization and sentence classification), joint training can lead to negative transfer (Liu et al., 2019). This work adopts a modular strategy: each module is trained independently, which ensures independent hyperparameter optimization, eliminates negative transfer, and allows for the replacement of individual modules. The pipeline has been enhanced with a deterministic post-processor (inversion algorithm) that ensures the semantic correctness of arguments for analytical tags.

### 2.4 Linguistic foundations of the taxonomy

The annotation scheme used in this work follows standard descriptions of Kazakh and Turkic morphosyntax. Causal subordination in Turkic languages is canonically realized through two principal strategies, traditionally referred to as the synthetic and analytic types (Johanson, 2021; Erdal, 2004; Soltanbekova and Utebaeva, 2022; Balakayev, 1959; Tukeshova, 2023).

The synthetic strategy nominalizes the dependent clause through a participial or action-nominal suffix (−ğan/−atyn/−u) and marks it with a case ending—most commonly the ablative −dan expressing source/cause, but also −üşın (benefactive) and forms involving the abstract-noun derivational suffix −dyq. Productive combinations such as −ğandyqtan, −atyndyqtan, −ğan soñ, and −ğany üşın constitute the inventory we refer to as Tv-forms.

The analytic strategy uses free morphemes—conjunctions and discourse connectives such as sebebı “because,” öitkenı “since,” sondyqtan “therefore,” sol sebeptı “for that reason.” A key syntactic fact, noted in descriptive grammars of Kazakh and other Turkic languages (Soltanbekova and Utebaeva, 2022; Balakayev, 1959), is that these connectives occupy a fixed interclausal position: the consequence precedes the marker and the cause follows it. This property is what motivates our positional-inversion algorithm.

The 16 Tv-form classes used in Module M3 correspond to productive combinations of a participial or action-nominal stem, an optional abstract nominalizer −dyq, and a causal case ending. Our threshold of ≥20 corpus occurrences excludes long-tail and unproductive forms while retaining all combinations attested as productive in modern written Kazakh; the resulting inventory matches the morphological descriptions given in Soltanbekova and Balakayev (Soltanbekova and Utebaeva, 2022; Balakayev, 1959).

The third category, ANALYTICO-SYNTHETIC, captures constructions in which a synthetic Tv-form co-occurs with an analytic connective—for example, sondyqtan introducing a clause that itself carries −ğandyqtan. Mixed strategies of this kind have also been widely discussed in Turkological studies on clause linkage and syntactic connectivity. As noted by Lars Johanson, Turkic languages frequently demonstrate hybrid structural patterns in which inherited Turkic forms coexist with later contact-induced and analytically developed constructions (Johanson, 2021). The smaller frequency of this class in our corpus (18.8%) and its comparatively lower F1 (0.893) are consistent with its status as a marked, internally heterogeneous category.

## 3 Methodology

### 3.1 Case description

The corpus was designed specifically for this project. The sources included journalistic, popular science, official business, and literary texts in the Kazakh language. Total volume: 3,223 sentences, each of which contains at least one marked causal relationship. The annotation was performed across four dimensions (Table 2) by two expert linguists, with any discrepancies subsequently resolved. The inter-rater reliability coefficient (Cohen’s *κ*) was 0.87 for clause segmentation, 0.91 for Tv forms, and 0.89 for construction type (Model Group). Any disagreements were resolved by the consensus of a third expert. The samples were divided into a training set (80%) and a test set (20%) based on stratification by structural type. The statistics for the corpus are shown in Table 2 (see Table 2).

**Table 2:** Statistics for the annotated corpus (*n* = 3,223), broken down by class and sample.

Class/type	Total	Training (80%)	Test (20%)	Percentage
CAUSE_CLAUSE	3,223	2,578	645	100
EFFECT_CLAUSE	3,183	2,546	637	98,8
MARKER	3,223	2,578	645	100
MG: SYNTHETIC	1,396	1,117	279	43,3
MG: ANALYTIC	1,222	978	244	37,9
MG: ANALYTICO-SYNTHETIC	605	484	121	18,8
Tv-forms (classes with ≥20 examples)	16	–	–	–
Total sentences	3,223	2,578	645	100

### 3.2 A taxonomy of Tv forms and types of syntactic constructions

Building on the linguistic foundations of Section 2.4, we operationalise Kazakh causal markers along two dimensions: construction type at the syntactic level (Module M4, three classes) and Tv-form at the morphological level (Module M3, 16 classes).

Construction type (Model Group, M4). The three values follow the established Turkological synthetic/analytic split, extended with a mixed category. Class-membership criteria:

- SYNTHETIC (*n* = 1,396; 43.3%). The dependent clause is nominalised by a participial or action-nominal suffix and bears a causal case ending. No free conjunction or connective is present. Following Soltanbekova and Utebaeva (2022) and Balakayev (1959), we treat as members of this class all combinations of a nominalised verb stem with a productive causal case (−dan, −üşın, −ğa dejin, etc.). Diagnostic: the marker is a bound morpheme that cannot be separated from the verb stem.
- ANALYTIC (*n* = 1,222; 37.9%). The marker is a free morpheme—a conjunction or discourse connective (sebebı, öitkenı, sondyqtan, sol sebeptı). Diagnostic: the marker can be removed from the verb without affecting the verb’s morphological form. Per Nevskaya et al. (2021), these markers occupy the interclausal slot EFFECT–MARKER–CAUSE and require the inversion procedure described in Section 3.3.
- ANALYTICO-SYNTHETIC (*n* = 605; 18.8%). The dependent clause carries a synthetic Tv-form and is additionally introduced or anchored by an analytic connective. Diagnostic: both a bound causal suffix and a free causal connective are present in the same construction. This category is corpus-attested in our data and has, to our knowledge, not previously been formalized as an annotation target.

#### 3.2.1 Tv-forms (M3)

The 16 classes encode distinctions along three morphological dimensions:

- participial (−ğan) vs. action-nominal (−u) vs. future-participial (−atyn) stem;
- presence or absence of the abstract-noun derivational suffix −dyq;
- causal case ending (−dan ablative, −üşın benefactive, −ğa dejin terminative, etc.).

#### 3.2.2 Clarification of the relationship between modules M3 and M4

Modules M3 and M4 are separated because they represent two different levels of linguistic analysis. Module M3 operates at the morphological and morphosyntactic level. It classifies the specific Tv-form or causal marker realization used in the sentence, for example *Tv = ğandyqtan, Tv = ğan soñ, Tv = mai/mei*, or other productive causal marker patterns. Thus, M3 answers the question of how causality is morphologically encoded.

Module M4 operates at a higher syntactic-constructional level. It classifies the entire causal construction into one of three broader structural groups: SYNTHETIC, ANALYTIC, or ANALYTICO-SYNTHETIC. Thus, M4 answers the question of which structural strategy is used to express causality in the sentence.

Although the two modules are related, they are not redundant. A single structural group may include several Tv-form realizations, while morphologically similar forms may receive different structural interpretations depending on the syntactic environment. For this reason, the separation of M3 and M4 allows the model to preserve fine-grained morphological information while also capturing higher-level syntactic generalizations. This modular distinction improves interpretability and reduces possible interference between form-level and construction-level classification tasks.

Table 3 lists each class with a representative example and the morphological gloss. The inclusion threshold of ≥20 corpus occurrences excludes *ad hoc* and unproductive forms while preserving combinations that are systematically productive in modern written Kazakh and Turkic languages, particularly Turkish, as described in the inventories proposed by Göksel (Göksel and Kerslake, 2025) and Balakayev (Balakayev, 1959).

**Table 3:** Annotation scheme: four modules, labels, and examples.

Module	Tags	Description	Example
M1—Markers	O, B-MARKER, I-MARKER	Lexical markers of causality (Tv-forms and analytical constructions)	Because, since, since, because
M2—Clauses	O, B-CAUSE, I-CAUSE, B-EFFECT, I-EFFECT	Segmentation of the antecedent and consequent clauses according to the BIO scheme	[… because]CAUSE [there is freedom.]EFFECT
M3—Tv-form	16 classes (categorical)	Morphological/syntactic type of marker (suffixal or analytical)	Tv-form; [(N1) since (N1)]; Tv = p; Tv = m; …
М4—Model Group	SYNTHETIC, ANALYTIC, ANALYTICO-SYNTHETIC	Type of syntactic structure of the marker (3 classes)	SYNTHETIC (suffix), ANALYTIC (postposition/conjunction), ANALYTICO-SYNTHETIC (mixed)

### 3.3 BIO diagram and data preprocessing

The BIO circuit is used for token marking tasks (modules M1 and M2). M1 uses the tags {O, B-MARKER, I-MARKER}; M2 uses the tags {O, B-CAUSE, I-CAUSE, B-EFFECT, I-EFFECT}. The alignment of the tokenization with the KazBERT tokenizer is achieved through offset mapping. For a token *tᵢ* with the range [*sᵢ*, *eᵢ*), the label *lᵢ* is defined as:


$$\begin{array}{c} l_{i}=B-X,\text{if}\;s_{i}=\text{start}({\text{span}}_{X})\\ l_{i}=I-X,\text{if}\;s_{i}\neq \text{start}({\text{span}}_{X})\wedge [s_{i},e_{i})\subset {\text{span}}_{X}\\ l_{i}=0,\text{otherwise} \end{array}$$

Where X ∈ {MARKER, CAUSE, EFFECT}, and ${\text{span}}_{X}$ is the annotated span of the corresponding class. For sequence classification tasks (M3, M4), the input is the full text of the sentence; tokenization is performed using WordPiece with max_length = 128.

### 3.4 Positional inversion algorithm

Analytic explanatory markers in Kazakh may occur in an interclausal position where the surface order follows the pattern EFFECT–MARKER–CAUSE. This order differs from the canonical semantic representation of causality, in which the cause precedes or determines the effect. Therefore, a deterministic positional inversion post-processor is included in the pipeline to normalize the semantic direction of analytic causal constructions when necessary.

The positional inversion rule is applied to analytic explanatory markers that may occur between the effect and the cause clause. In the present implementation, the marker set is defined as:


$$M\_\text{expl}=\{\text{sebebi},\ddot{o}\text{itkeni},\text{sondyqtan},\mathrm{sol}\;\text{sebepti}\}$$

Where sebebi means “because,” öitkeni means “since/because,” sondyqtan means “therefore,” and sol sebepti means “for that reason.” These markers may indicate that the surface order of the construction follows the pattern EFFECT–MARKER–CAUSE. In such cases, the post-processor can restore the correct semantic direction by swapping the predicted cause and effect arguments when the positional condition is met:

if marker ∈ M_expl ∧ pos(CAUSE) < pos(MARKER) < pos(EFFECT):

CAUSE ↔ EFFECT

Here, pos(·) denotes the position of the initial character of the corresponding span in the input string. The rule is applied after the outputs of M1 and M2 are generated and before the final structured JSON output is formed.

Importantly, this component is applied after the neural prediction stage. Therefore, it does not replace the M2 clause segmentation model, but functions as a deterministic safety and normalization mechanism for analytic causal constructions. Its purpose is to reduce the risk of systematic argument-order errors caused by the mismatch between surface clause order and semantic cause–effect direction.

### 3.5 System architecture

**Figure 2:**
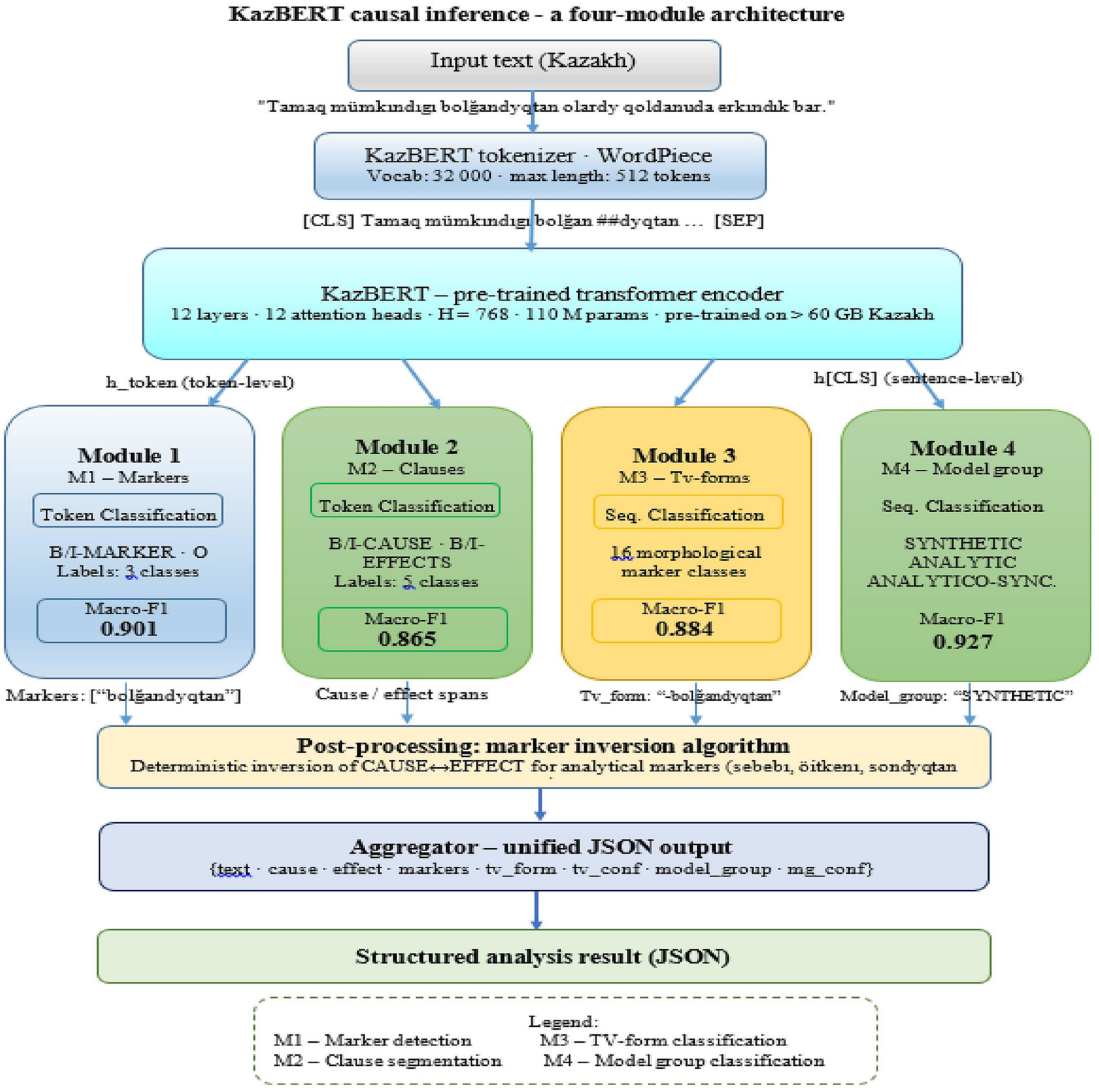
A four-module system architecture based on KazBERT.

The proposed four-module architecture is shown in Figure 2. All four modules are initialized independently using KazBERT parameters (BERT-base: 12 layers, 12 attention heads, H = 768, 110 M parameters) and are fine-tuned on task-specific data (see Figure 1).

Modules M1 and M2 implement token marking. A linear classifier with Softmax is applied to the hidden state of each token $h_{i}\in R_{v}$ from the final layer of the encoder:


$$P(y_{i}\;|\;x_{i})=\text{softmax}(W_{\mathrm{tok}}\cdot h_{i}+b_{\mathrm{tok}}),W_{\mathrm{tok}}\in R^{\left(|C|\times H\right)}$$

Modules M3 and M4 implement sentence classification based on the token’s hidden state [CLS]:


$$P\;(y\;|\;x)=\text{softmax}(W_{\mathrm{CLS}}\cdot h[\mathrm{CLS}]+b\_\mathrm{cls}),W_{\mathrm{CLS}}\in R^{\left(|K|\times H\right)}$$

Where *K* is the set of classes (Tv-forms or construction types). The loss function for both types of tasks is standard cross-entropy:


$$L=-(1/N)\sum \_i\sum \_(y_{k})\;y_{k}\cdot \log\;P\;(y_{k}\;|\;x_{i})$$

### 3.6 Training hyperparameters

Table 4 contains a complete set of hyperparameters for the four modules. All experiments were performed on an NVIDIA GPU with FP16 mixed-precision support. Early stopping strategy: saving the checkpoint with the lowest eval_loss value (see Table 4).

**Table 4:** Training hyperparameters for the four modules.

Hyper parameter	M1 (markers)	M2 (clauses)	M3 (Tv-form)	М4 (model group)
Base model	KazBERT	KazBERT	KazBERT	KazBERT
Task type	Token-CLS	Token-CLS	Seq-CLS	Seq-CLS
Learning rate (lr)	2 × 10^−5^	2 × 10^−5^	2 × 10^−5^	2 × 10^−5^
Batch size (train/eval)	8/8	8/8	16/16	16/16
Number of epochs	5	5	8	8
Regularization (weight_decay)	0.01	0.01	0.01	0.01
Maximum sequence length	128	128	128	128
Mixed precision (FP16)	Yes	Yes	Yes	Yes
Number of tags/classes	3	5	16	3
Named entity recognition (NER)/strategy	“First”	“First”	Argmax	Argmax

### 3.7 Evaluation metrics

For token labeling tasks (M1, M2), the primary metric is macro-F1, which accounts for the impact of class imbalance:


$$F1_{\text{macro}}=\frac{1}{|C|}\sum_{c\in C}\frac{2P_{c}R_{c}}{P_{c}+R_{c}}$$

Where $P_{c}=\frac{{\mathrm{TP}}_{c}}{{\mathrm{TP}}_{c}+{\mathrm{FP}}_{c}}$ is precision, and $R_{c}=\frac{{\mathrm{TP}}_{c}}{{\mathrm{TP}}_{c}+{\mathrm{FN}}_{c}}$ is recall for class c. For sequence classification tasks (M3, M4), Accuracy is additionally used to reflect the proportion of correctly classified sentences. All metrics are calculated using a fixed test sample (645 sentences, 20% of the corpus).

## 4 Results

### 4.1 Analysis of the body structure

**Figure 3:**
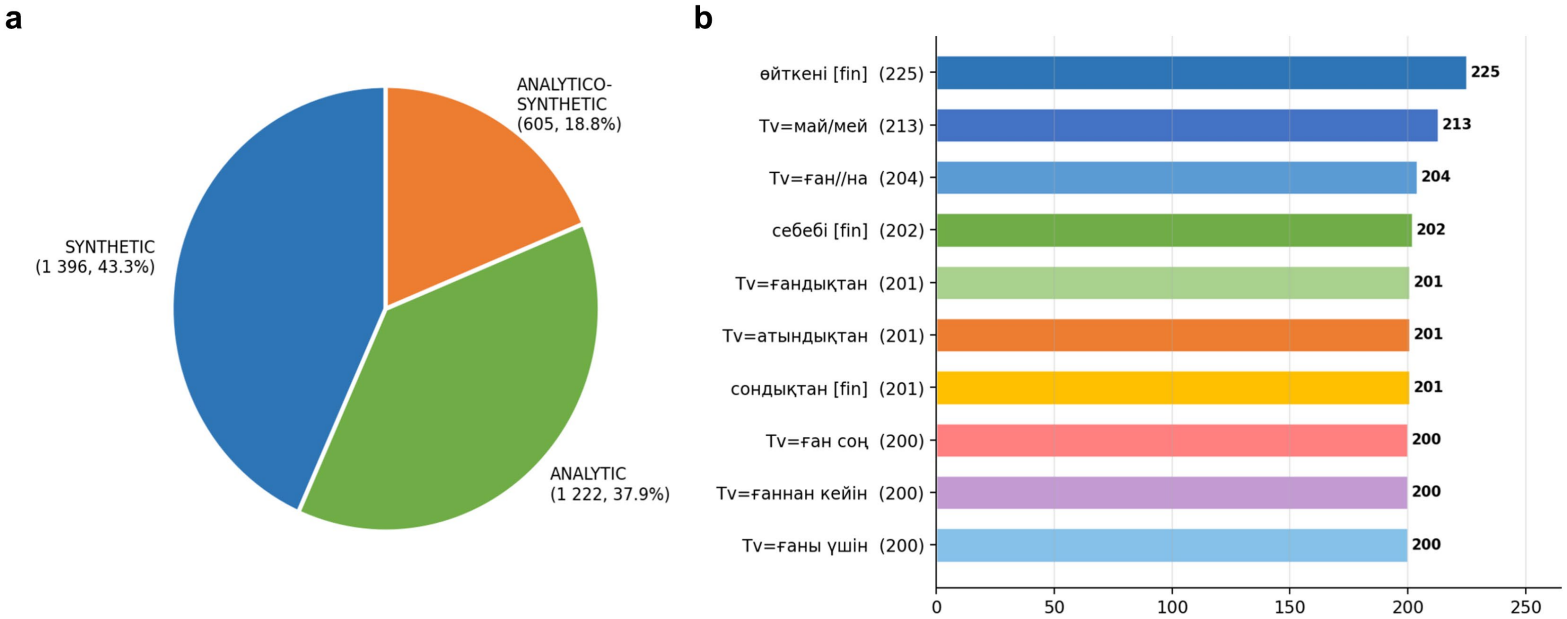
Distribution of structural types (a) and the top 10 Tv-forms across 16 categories (b).

The types of syntactic constructions are represented in the corpus in a relatively even distribution: SYNTHETIC—43.3%, ANALYTIC—37.9%, ANALYTICO-SYNTHETIC—18.8% (Figure 3a). The comparable frequency of synthetic and analytical strategies is a new corpus-based observation indicating that both strategies are productive and equally valid in the written Kazakh language. The distribution of Tv-forms across the 16 classes is uniform: ranging from 183 (Tv = p/e/i) to 225 examples ([(N1) öitkenı (N1)]), which ensures stable training for all classes (see Figure 3b).

### 4.2 Model comparison

Table 4 presents the evaluation results for three models across four subtasks on the test set (*n* = 645). The best values for each metric are highlighted in bold. Figure 3 provides a graphical comparison based on macro-F1 (see Table 5).

**Table 5:** Results of the evaluation on the test sample (*n* = 645).

Task	Model	Precision	Recall	F1 macro	Accuracy
М1: Markers	KazBERT	**0.912**	**0.891**	**0.901**	**0.946**
	XLM-R	0.875	0.852	0.863	0.913
	mBERT	0.851	0.828	0.839	0.891
М2: Clauses	KazBERT	**0.879**	**0.851**	**0.865**	**0.928**
	XLM-R	0.843	0.816	0.829	0.896
	mBERT	0.819	0.792	0.805	0.872
M3: Tv-form (16-bit)	KazBERT	**0.897**	**0.871**	**0.884**	**0.931**
	XLM-R	0.863	0.840	0.851	0.899
	mBERT	0.839	0.814	0.826	0.876
М4: Model Group classification (3 classes)	KazBERT	**0.934**	**0.920**	**0.927**	**0.949**
	XLM-R	0.899	0.884	0.891	0.918
	mBERT	0.875	0.861	0.868	0.893

The best values are highlighted in bold.

**Figure 4:**
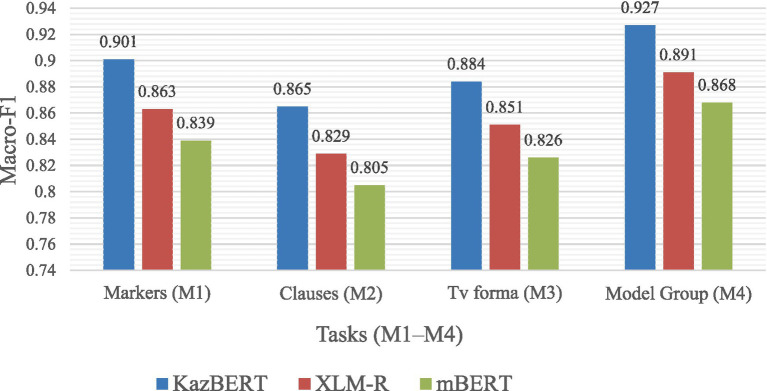
Comparison of model performance across four tasks (Macro-F1).

KazBERT outperforms both baseline solutions by a statistically significant margin across all four subtasks. The improvement of macro-F1 relative to XLM-RoBERTa ranges from 3.3 percentage points (metrics) to 3.8 percentage points (Tv-form). Compared to mBERT, the improvement ranges from 4.5 to 6.2 percentage points (see Figure 4).

The highest absolute F1 score of 0.927 was achieved in the structure type classification task (M4), indicating that the SYNTHETIC, ANALYTIC, and ANALYTICO-SYNTHETIC classes are well-separated in the KazBERT representation space. The clause segmentation task (M2) yields the lowest absolute F1 score of 0.865, which can be attributed to the difficulty of accurately identifying the boundaries of nested clauses across all three types of constructions simultaneously.

To gain a fuller understanding of the experimental results, the effectiveness of the proposed model can be explained through the comparative analysis presented in Table 1 and Figure 1. The results confirm that the proposed four-module architecture provides a balanced combination of performance, interpretability and adaptation to resource-constrained conditions, which is not the case with existing approaches.

### 4.3 Error analysis

#### 4.3.1 Ablation analysis of the positional inversion module

To evaluate the contribution of the positional inversion module, we conducted an ablation analysis on the held-out test set. The full annotated corpus contains 3,223 Kazakh causal sentences, which were divided into 2,578 training sentences and 645 test sentences. The positional inversion rule is not applied to all causal constructions; it is relevant only for analytic explanatory constructions in which the surface order may follow the pattern EFFECT–MARKER–CAUSE.

In the held-out test set, 187 analytic-marker cases were identified and evaluated. The same trained M2 clause segmentation model was tested under two conditions. First, the raw neural output was evaluated without applying the positional inversion rule. Second, the same predictions were evaluated after applying the deterministic positional inversion post-processor. The model was not retrained for this experiment; only the post-processing step was enabled or disabled. This design isolates the specific contribution of the positional inversion rule to the final cause–effect argument ordering.

The ablation analysis showed that the trained clause segmentation model already achieved 100.0% correct argument ordering on the 187 analytic-marker test cases before applying the positional inversion rule. After applying the positional inversion post-processor, the result remained 100.0%, corresponding to a 0.0 percentage-point change (Table 6).

**Table 6:** Ablation analysis of the positional inversion module.

Evaluation setting	Description	Covered cases	Correct argument order	Remaining errors
Without positional inversion	Raw output of the trained M2 clause segmentation model	187	100.0%	0.0%
With positional inversion	M2 output with deterministic positional inversion post-processing	187	100.0%	0.0%
Difference	Change after applying the post-processor	–	+0.0 percentage points	–

These results indicate that the trained M2 model had already learned the semantic cause/effect roles in the evaluated analytic-marker cases. Therefore, the positional inversion component does not increase aggregate test-set accuracy in this particular evaluation. Nevertheless, it is retained as a deterministic safety and normalization mechanism for analytic EFFECT–MARKER–CAUSE constructions. This module explicitly encodes a known syntactic property of Kazakh analytic causal constructions and increases the robustness and interpretability of the complete system.

A qualitative analysis of incorrect predictions revealed several consistent patterns. In the clause segmentation task (M2), approximately 58% of errors occur in sentences containing embedded participial phrases or multiple Tv forms: the model tends to blur the boundaries between adjacent clauses. Errors tend to occur most frequently in analytico-synthetic constructions, where the boundary between cause and effect is linguistically the least clear-cut. In the task of classifying Tv forms (M3), the greatest difficulty lies in distinguishing between morphologically similar pairs: *Tv = ğan//na* vs. *Tv = ğan//nan* (differing only in case inflection) and the pairs of analytical connectives: [(N1) *sondyqtan* (N1)] vs. [(N1) *sol sebeptı* (N1)]. These two pairs account for about 34% of M3 errors. In the structure classification task (M4), errors are concentrated in the ANALYTICO-SYNTHETIC category (F1 = 0.893, compared to 0.941 for SYNTHETIC and 0.946 for ANALYTIC), which corresponds to its smaller size and greater linguistic heterogeneity.

### 4.4 Example of the KazCausal pipeline in operation

To illustrate how the system works, we consider the following sentence:

*Qoğamdyq pıkırdıñ polärizasialanatynyna alañdadyq, medialyq keñıstıkte radikaldy ritorika küşeidı.*

(Translation: *We were concerned about the polarization of public opinion, which has led to an increase in radical rhetoric in the media*.)

The system returns as: {cause: “qoğamdyq pıkırdıñ polärizasialanatynyna alañdadyq”; effect: “medialyq keñıstıkte radikaldy ritorika küşeidı”; markers: [“polärizasialanatynyna “]; Tv_form: “Tv = atyn//na, etın//ne, itın//ne, ityn//na, itın//ne”; Tv_confidence: 0.9950; model_group: “SYNTHETIC”; model_group_confidence: 0.9998}.

The system correctly identified the suffixal Tv form (−atynyna), determined that it was a SYNTHETIC construction, and established the boundaries of the causal and consequential clauses without using inversion (the marker occupies the CAUSE–MARKER position, which is characteristic of synthetic forms).

### 4.5 Web interface and input modes

In addition to the trained models, the proposed system was implemented as a KazCausal web application with a Kazakh-language user interface. The interface was designed to make the causal relation extraction pipeline accessible for practical use, demonstration, and qualitative inspection of model predictions.

The application supports three input modes. First, the user can analyze predefined example sentences embedded in the program. This mode is useful for testing and demonstrating typical Kazakh causal constructions. Second, the user can paste any Kazakh text into the text input field. In this mode, the system processes the entered text, identifies causal constructions, and returns structured annotations. Third, the user can upload an external TXT or CSV file, after which the system reads the file content and analyzes the text automatically.

**Figure 5:**
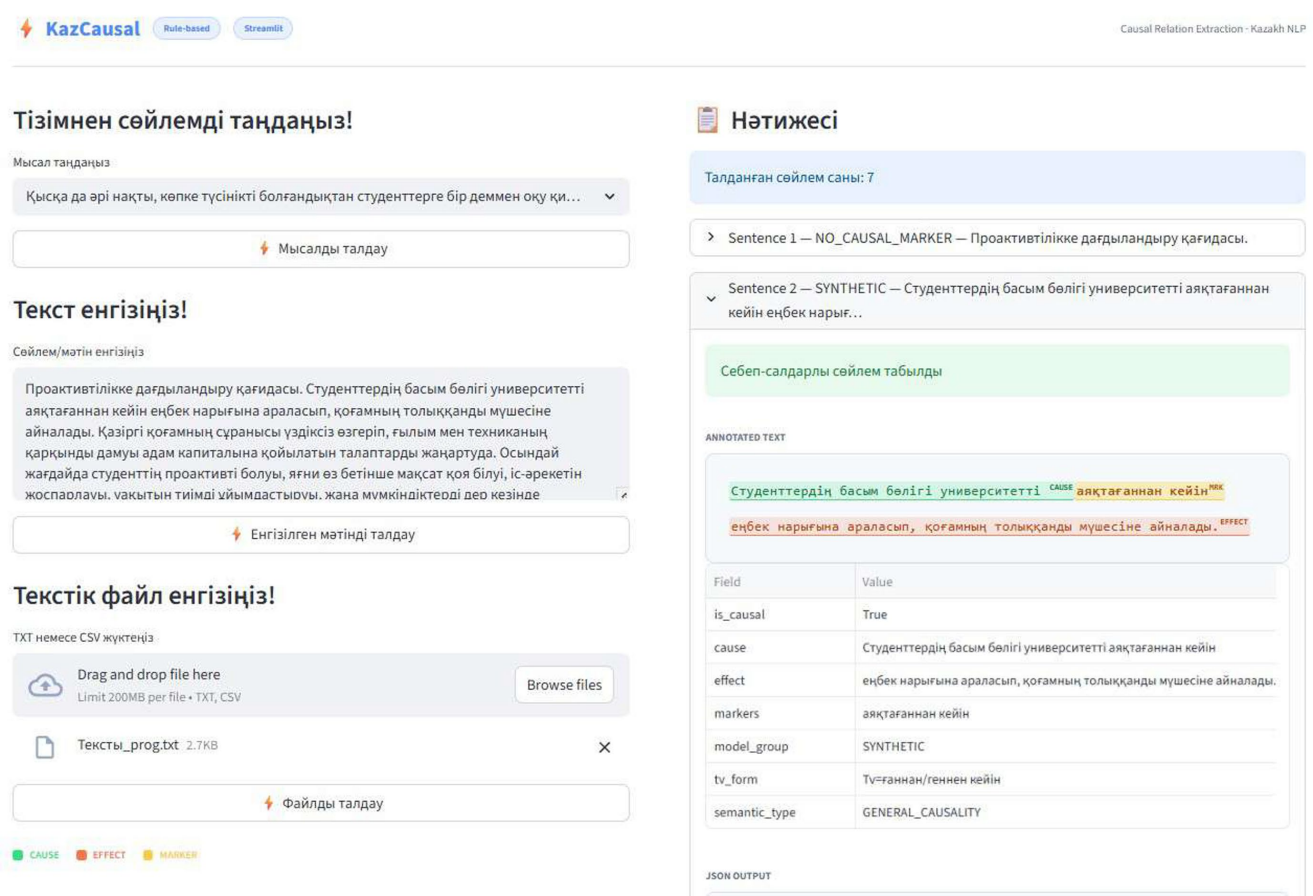
KazCausal web interface with three input modes: **(a)** analysis of predefined example sentences embedded in the program; **(b)** analysis of user-pasted Kazakh text; and **(c)** analysis of external TXT/CSV files. The output panel presents highlighted cause, effect, and marker spans together with Tv-form, construction type, semantic type, confidence scores, and structured JSON results.

As shown in Figure 5, the output panel displays the detected causal construction, including the cause span, effect span, causal marker, Tv-form, construction type, semantic type, confidence scores, and structured JSON output. The interface also visually highlights cause, effect, and marker spans using different colors, which improves interpretability and allows users to verify the model prediction.

It should be noted that the current implementation processes longer texts by identifying and analyzing sentence-level causal constructions. Therefore, the system can work with large pasted texts and uploaded files in a practical environment. However, cross-sentence and paragraph-level causal relation modeling remains outside the scope of the current version and is considered a future research direction.

## 5 Discussion

The results obtained allow us to draw several important conclusions. Firstly, pre-training on a Kazakh-language corpus gives the KazBERT model a consistent advantage across all four tasks. This is particularly evident in tasks requiring morphological generalization, namely in marker identification (M1) and the classification of Tv forms (M3). This result is consistent with the findings of other studies on agglutinative languages, in which language-specific models demonstrate superiority over multilingual models in processing complex morphological structures.

Secondly, the introduction of the fourth module—the classification of syntactic construction types (M4)—is an important scientific discovery from both linguistic and engineering perspectives. The observed distribution of 43.3%/37.9%/18.8% indicates that synthetic and analytical strategies in the written Kazakh language are productive to a similar extent. This is an important new finding in the study of the typological characteristics of Turkic languages.

Third, the positional inversion component functions as a deterministic safety and normalization mechanism for analytic EFFECT–MARKER–CAUSE constructions. The ablation analysis on 187 analytic-marker cases in the held-out test set showed that the trained M2 clause segmentation model achieved 100.0% correct cause–effect argument ordering both before and after applying the positional inversion post-processor. This indicates that the neural model had already learned the semantic cause–effect roles in the evaluated cases. Nevertheless, the positional inversion rule is retained because it explicitly encodes a known syntactic property of Kazakh analytic causal constructions and helps prevent potential argument-order errors when raw model outputs follow surface clause order rather than semantic direction.

These results are also confirmed by the comparative analysis presented in Table 1 and Figure 1. As shown in the heatmap visualization, the proposed architecture demonstrates balanced performance across several key metrics, such as performance, interpretability, multilingualism, and adaptation to resource-constrained environments. Whilst existing methods often focus on optimizing only a single metric, the proposed approach combines these properties within a single system.

Error analysis results showed that the remaining difficulties are mainly related to complex syntactic structures. In particular, this applies to embedded clauses and morphologically similar Tv forms. Such difficulties are linked to the natural characteristics of agglutinative languages, as even a small difference in morphological changes can significantly affect semantic interpretation.

Several limitations condition the generalizability of our findings.

- Sentence-level scope. A further limitation of the current framework is that it primarily focuses on sentence-level causal relation extraction. In the present version, cause and effect spans are detected mainly within the boundaries of a single sentence. However, in real scientific and academic discourse, causal relations may extend across multiple sentences or paragraphs. For example, a cause may be introduced in one sentence, while its consequence may be explained in the following sentence or in a later paragraph. The implemented KazCausal application partially addresses the practical processing of longer texts by supporting three input modes: predefined example sentences, pasted free text, and external TXT/CSV files. In these modes, the system can scan longer Kazakh texts and identify sentence-level causal constructions. Nevertheless, the current model does not yet perform full discourse-level linking of causes and effects across sentence boundaries. Future work will therefore focus on extending the framework to discourse-level causal relation extraction by incorporating paragraph-level context, inter-sentential dependencies, coreference resolution, and discourse-aware transformer representations. Such an extension will allow the system to identify causal relations not only within individual sentences but also across larger textual units.
- Corpus size. At 3,223 sentences, our corpus is small by contemporary NLP standards. Stratified sampling and per-module training mitigate overfitting, but we cannot fully rule out that some of the reported gains reflect memorization of frequent marker forms rather than genuine generalization. This concern is sharpest for the ANALYTICO-SYNTHETIC class (*n* = 605), whose lower F1 (0.893) is consistent with both its smaller size and its greater linguistic heterogeneity.
- Genre and modality. All texts are written and drawn from journalistic, popular-science, official-business, and literary registers. Spoken Kazakh, social-media text, and domain-specific scientific/technical writing are not represented. The 43.3%/37.9%/18.8% distribution of construction types should accordingly be read as a property of written Kazakh in these registers, not of Kazakh in general; spoken Kazakh in particular is known to favor analytic strategies more heavily.
- Annotation pool. Two expert linguists performed the annotation, with disagreements adjudicated by a third. Cohen’s *κ* values of 0.87–0.91 indicate substantial agreement but leave room for systematic bias that a larger annotator pool would expose. Inter-annotator reliability for Module M1 (marker spans) is not separately reported in the present version and should be added.
- Long-tail Tv-forms. Our ≥20-example threshold excludes rarer suffixal patterns. The reported macro-F1 on M3 therefore reflects performance on productive forms only and may overstate accuracy on a fully open Tv-form inventory.
- Out-of-distribution evaluation. All evaluation is performed on a held-out 20% slice of the same corpus. No external benchmark or out-of-domain test is yet available, and we do not yet evaluate transfer to closely related Turkic languages.

Future work will address these limitations by extending the corpus to spoken and informal registers, increasing the annotator pool and reporting per-module κ, lowering the Tv-form inclusion threshold once enough examples are available, and constructing an external Kazakh-language benchmark for causal extraction.

Overall, the proposed approach not only improves accuracy but also ensures the interpretability and adaptability of the results, making it the preferred solution for morphologically complex and low-resource languages.

## 6 Conclusion

This paper presents a four-module neural architecture for the automatic identification and classification of causal relationships in the Kazakh language based on the KazBERT model. The proposed method combines tasks such as marker identification, clause segmentation, classification of Tv-forms, and syntactic construction type identification into a single, yet modular structure.

Experimental results demonstrated that the proposed model consistently outperforms multilingual baseline models across all its sub-tasks, with a macro-F1 score reaching 0.927. The results showed that language-adapted pre-training and a modular architecture are effective for morphologically complex and low-resource languages. Furthermore, the positional inversion algorithm was evaluated through an ablation analysis and is retained as a deterministic safety and normalization mechanism for analytic EFFECT–MARKER–CAUSE constructions. Although the trained M2 clause segmentation model already achieved correct argument ordering on the evaluated analytic-marker test cases, the rule explicitly encodes a known syntactic property of Kazakh analytic causal constructions and helps prevent potential argument-order errors in practical use.

The research makes an important contribution both methodologically and linguistically. A methodologically interpretable and extensible architecture is proposed, whilst linguistically, new corpus-based findings on the prevalence and productivity of causal constructions in the Kazakh language have been obtained.

However, the study has certain limitations. The current model primarily focuses on sentence-level causal relations and does not yet fully capture causal links that extend across multiple sentences or paragraphs. Future research will therefore explore discourse-level causal modeling, larger and more diverse datasets, and the integration of the modular architecture with multi-task learning and large language models.

Overall, the proposed approach provides an effective foundation for the automatic identification of causal relationships in morphologically complex and under-resourced languages, such as Kazakh.

## Data Availability

The datasets presented in this study can be found in online repositories. The names of the repository/repositories and accession number(s) can be found below: The dataset, source code and trained models are available at: https://github.com/ParserRn/KazCausal.

## References

[ref1] Balakayev M. B. (1959). Modern Kazakh Language: Syntax of Word Combinations and the Simple Sentence. Publishing House of the Academy of Sciences of the Kazakh SSR.

[ref2] Chen Y. Zhao H. Li Z. Zhou G. (2022). ERGO: event relational graph transformer for document-level event causality identification. Proceedings of COLING, 2118–2128. doi: 10.48550/arXiv.2204.07434

[ref3] Conneau A. Khandelwal K. Goyal N. Chaudhary V. Wenzek G. Guzmán F. . (2020). Unsupervised cross-lingual representation learning at scale. Proceedings of ACL, 8440–8451. doi: 10.18653/v1/2020.acl-main.747

[ref4] Crawshaw M. (2020). Multi-task learning with deep neural networks: a survey. arXiv 2020, arXiv:2009.09796.

[ref5] Dasgupta T. Saha R. Dey L. Naskar A. (2018). Automatic extraction of causal relations from text using linguistically informed deep neural networks. Proceedings of EMNLP, 306–316. doi: 10.18653/v1/D18-1031

[ref6] Devlin J. Chang M.-W. Lee K. Toutanova K. (2019). BERT: pre-training of deep bidirectional transformers for language understanding. Proceedings of NAACL-HLT, 4171–4186. doi: 10.18653/v1/N19-1423

[ref7] Erdal M. (2004). A Grammar of Old Turkic, vol. 3 Leiden; Boston; Koln: Brill.

[ref8] Gao L. Choubey P. K. Huang R. (2019). Modeling document-level causal structures for event causal relation identification. Proceedings of NAACL-HLT, 1808–1817. doi: 10.18653/v1/N19-1180

[ref9] Girju R. (2003). Automatic detection of causal relations for question answering. ACL Workshop on Multilingual Summarization and QA, 76–83. doi: 10.3115/1119312.1119322

[ref10] Girju R. Nakov P. Nastase V. Szpakowicz S. Turney P. Yuret D. . (2007). “SemEval-2007 task 4: classification of semantic relations between nominals,” in Proceedings of the 4th International Workshop on Semantic Evaluations 2007, (), 13–18.

[ref11] Göksel A. Kerslake C. (2025). Turkish: A Comprehensive Grammar. London and New York: Routledge.

[ref12] Heindorf S. Scholten Y. Wachsmuth H. Akhtar N. Potthast M. (2020). CauseNet: towards a causality graph extracted from the web. Proceedings of CIKM, 3023–3030. doi: 10.1145/3340531.3412763

[ref13] Johanson L. (2021). Turkic (Cambridge Language Surveys). Cambridge: Cambridge University Press.

[ref14] Khassanov Y. Mussakhojayeva S. Mirzakhmetov A. (2021). A crowdsourced open-source Kazakh speech Corpus and initial speech recognition baseline. Proceedings of EACL, 75–80. doi: 10.18653/v1/2021.eacl-demos.10

[ref15] Liu X. He P. Chen W. Gao J. (2019). Multi-task deep neural networks for natural language understanding. Proceedings of ACL, 4487–4496. doi: 10.18653/v1/P19-1441

[ref16] Makhambetov O. Makazhanov A. Yessenbayev Z. (2013). Matrassulova. Proceedings of Corpus Linguistics: D. Assembling the Kazakh Language Corpus.

[ref17] Nevskaya I. A. (2021). Participial Constructions in Turkic Languages: A Functional-Semantic Approach. Russia: Science: Novosibirsk.

[ref18] Nevskaya I. A. Ozonova A. A. Tazranova A. R. Fedina N. N. (2021). Semantics and scope of reference of first-person non-singular imperative forms. Critiq. Semi. 2, 210–233. doi: 10.25205/2307-1737-2021-2-210-233

[ref19] Özateş Ş. B. Kurfali M. Toprak C. (2022). Causal relation annotation in Turkish: challenges and initial findings. Proceedings of LREC, 5118–5126.

[ref20] Soltanbekova A. A. Utebaeva E. A. (2022). Features of the use of function words in “Kazakh grammar” based on Semasiological and Onomasiological approaches. Tiltanym 86, 98–106. doi: 10.55491/2411-6076-2022-2-95-103

[ref21] Tan H. Chen X. Feng J. (2022). Document-level event causality identification via graph information interaction learning. Proceedings of ACL, 1, 3552–3562. doi: 10.18653/v1/2022.acl-long.248

[ref22] Tukeshova N. M. (2023). Antonymic component phraseological units in English and Kazakh languages (comparative analysis). Actual Problems of Linguistics 110, 6–12. doi: 10.31489/2023Ph2/6-12

[ref23] Yang B. Mihalcea R. (2022). Causal reasoning about entities and events with attention networks. Findings of EMNLP, 2510–2521. doi: 10.18653/v1/2022.findings-emnlp.186

[ref24] Yeshpanov R. Khassanov Y. Varol H. A. (2022). KazBERT: Kazakh language model for downstream tasks. arXiv 2022, arXiv:2205.12445.

[ref25] Zhu C. Chen M. Han S. Gao J. Liu J. (2024). Causal reasoning in the era of large language models: a survey. IEEE Trans. Neural Networks Learn. Syst. 35, 1–18. doi: 10.1109/TNNLS.2023.3261203

